# Paper-Based Test for Rapid On-Site Screening of SARS-CoV-2 in Clinical Samples

**DOI:** 10.3390/bios11120488

**Published:** 2021-11-30

**Authors:** Wen Ren, Joseph Irudayaraj

**Affiliations:** 1 Department of Bioengineering, University of Illinois at Urbana-Champaign, Urbana, IL 61801, USA; wenren@illinois.edu; 2 Biomedical Research Center, Mills Breast Cancer Institute, Carle Foundation Hospital, Urbana, IL 61801, USA; 3 Micro and Nanotechnology Laboratory, University of Illinois at Urbana-Champaign, Urbana, IL 61801, USA; 4 Cancer Center at Illinois (CCIL), University of Illinois at Urbana-Champaign, Urbana, IL 61801, USA

**Keywords:** lateral flow assay, magnetic focus enhancement, colorimetric detection, SARS-CoV-2 monitoring, clinical samples

## Abstract

Detection methods for monitoring infectious pathogens has never been more important given the need to contain the spread of the COVID-19 pandemic. Herein we propose a highly sensitive magnetic-focus-enhanced lateral flow assay (mLFA) for the detection of SARS-CoV-2. The proposed mLFA is simple and requires only lateral flow strips and a reusable magnet to detect very low concentrations of the virus particles. The magnetic focus enhancement is achieved by focusing the SARS-CoV-2 conjugated magnetic probes in the sample placed in the lateral flow (LF) strips for improved capture efficiency, while horseradish peroxidase (HRP) was used to catalyze the colorimetric reaction for the amplification of the colorimetric signal. With the magnetic focus enhancement and HRP-based amplification, the mLFA could yield a highly sensitive technology for the recognition of SARS-CoV-2. The developed methods could detect as low as 400 PFU/mL of SARS-CoV-2 in PBS buffer based on the visible blue dots on the LF strips. The mLFA could recognize 1200 PFU/mL of SARS-CoV-2 in saliva samples. With clinical nasal swab samples, the proposed mLFA could achieve 66.7% sensitivity and 100% specificity.

## 1. Introduction

The effect of the recent COVID-19 pandemic has brought forth tremendous infections and deaths among other negative influences on human life and economics. The increasing number of vaccinations brings a shimmering hope to control the pandemic. The SARS-CoV-2 virus, which causes COVID-19, exhibits a high mutation rate and generated multiple variants. Some of the variants could spread faster, induce more severe illness and even breakthrough infection with the vaccinated population, such as the Delta variant. Meanwhile, it should be noted that the list of the new variants of SARS-CoV-2 is on the rise. Therefore, in addition to the development of effective vaccines and virus mutation, technologies for infection monitoring to control the spread of SARS-CoV-2 is critical for saving lives and resources.

To monitor the spread of SARS-CoV-2, analytical methods that are easy to use are the cornerstone. Until now, several test strategies have been reported for the identification of SARS-CoV-2 or the diagnosis of COVID-19 infections. Briefly, these strategies are based on the SARS-CoV-2 virus itself or the antibodies generated by the immune system against the virus. The RNA of SARS-CoV-2 is the most common target for the identification of the virus. The highly specific sequences enables the recognition of different variants of SARS-CoV-2, while the chosen signature sections on the virus RNA could be dramatically amplified with various nucleic acid amplification techniques, such as reverse transcription-polymerase chain reaction (RT-PCR), reverse transcription loop-mediated isothermal amplification (RT-LAMP) and CRISPR/CAS [1,2,3,4,5,6,7]. Meanwhile, it is noted that some defects limit the use of these RNA-based virus tests: (i) the sample preparation, especially RNA extraction and amplification requires the use of laboratory facilities and trained operators, which are scarce in developing/under-developed countries, (ii) the corresponding sample collection and transport are labor-consuming and poses the additional risk of virus exposure, (iii) the cost of RNA-based tests assisted with amplification techniques would be unaffordable for low-income countries. It should be noted that according to the information from the World Health Organization (WHO) most of the variants of concern (VOC) were found in highly dense populated regions (mostly in under-developed countries) that lack test and medical care [8] The VOC from these hotbeds could spread worldwide. Therefore, test methods for the detection of SARS-CoV-2 that are practical and affordable for implementation in developing countries would be critical for timely monitoring and reporting of the CARS-CoV-2 infection. Furthermore, recent research indicated that RNA indicators could potentially give rise to false positives due to the integration of the viral RNA into the infected patients’ genome [9]. Another common route for the diagnosis of COVID-19 infection is to recognize the antibodies generated by the immune system against the infection by SARS-CoV-2. Due to the large number of antibodies generated, the detection methods based on this route could be performed without specific amplification. Various low-cost and simple detection methods targeting the antibodies have been reported [10,11,12]. However, the application of these methods and products are quite limited. There is a 2–3-week delay in the generation of antibodies from the time of infection, hence detection methods do not yield timely results. Therefore, it is believed that SARS-CoV-2 itself could be the better target for diagnosis and to track the spread of the virus. A technique that is highly sensitive and yet, practical, affordable, and rapid is the current need.

Compared to other signal readout techniques, lateral flow assay (LFA)-based strategies require only paper-based strips and do not require any additional instrumentation. LFA-based methods are practical and can be adapted for direct monitoring of SARS-CoV-2 with the necessary enhancement steps. There are two primary routes to enhance the detection sensitivity of LFA-based techniques: (i) to amplify the signal, and (ii) to improve the capture efficiency of the labeled targets on the LFA strip. The amplification of the LFA signal could be achieved with nanostructures that yield stronger extinction [13,14,15], gold or silver staining [16,17], enzymatic amplification [18,19], fluorescence [20,21], surface plasmon resonance or magnetic signal with corresponding readers [22,23]. In an LFA, the labeled targets flow through the microchannels in the strips along the sample solution and thus interact with the antibodies conjugated on the LF strips in a very limited time, resulting in a low capture efficiency which was estimated to be less than 1% [24]. By increasing the capture efficiency, more targets could be captured on the LF strips for enhanced detection. LF strips with wax pillar patterns or a sponge between the conjugate pad and the nitrocellulose membrane were utilized to reduce the flow speed on the strips to result in ~10-fold increase in sensitivity [25,26]. Other strategies include electrophoresis, termed as isotachophoresis (ITP), used for controlling the movement of the probe-labeled targets in the strip [24,27,28]. The ITP-based LFA could provide an enhancement in sensitivity of up to ~1000 folds. However, additional instruments for current control in the LF strips make the test system complex and less practical.

We propose an LFA-based detection strategy termed as magnetic-focus-enhanced lateral flow assay (mLFA) with greatly improved sensitivity while retaining the simplicity. In the mLFA test, magnetic focus enhancement was utilized to increase the capture efficiency of the magnetic-probe-labeled targets at the signal generation zone utilizing a magnet beneath the test zone of the LF strips. The magnetic field serves to reduce the flow speed of the magnetic probes and helps to concentrate the labeled targets in the microchannels of the LF strips. The capture efficiency is increased because of the concentrated target virus conjugated with the magnetic probe in the test zone and the reduced movement of the targets that enables a longer interaction time of target bound probes with the antibodies immobilized on the LF strips. Meanwhile, the visible signal in the mLFA is amplified with horseradish peroxidase (HRP)-catalyzed colorimetric reaction and the obtained blue spots could be recognized with the naked eye. By combining the magnetic focus enhancement and the HRP-based signal amplification, the mLFA has been shown to increase the sensitivity by as much as 10^6^-fold [29,30,31]. The mLFA has been shown to identify as low as ~2 bacterial cells [29]. Although SARS-CoV-2 viruses are much smaller than bacteria, with fewer receptor antigens, the magnetic focus enhancement could be a viable technology to achieve high detection sensitivity for SARS-CoV-2.

In this work, Fe_3_O_4_/Au core/shell magnetic nanoparticles were modified with antibodies specific to the target virus. The effect of the number of magnetic probes on the detection performance was also investigated. The detection capability of the proposed mLFA for SARS-CoV-2 monitoring was first demonstrated with serial concentrations of the target in PBS buffer. To align with the CDC RT-PCR requirements of 1200 cp/mL [32], the mLFA detection method was validated to detect as low as 1200 PFU/mL of SARS-CoV-2 positive control (ATCC^®^ VR-1986HK™, from American Type Culture Collection (ATCC), Manassas, VA, USA) in human saliva. Furthermore, utilizing clinical nasal swab patient samples, the mLFA for SARS-CoV-2 demonstrated a 66.7% sensitivity and 100% specificity.

## 2. Experimental Methods

### 2.1. Chemicals and Reagents

Ferrous chloride, ferric chloride, HAuCl_4_∙XH_2_O, sodium carbonate, and sodium citrate were obtained from Sigma Aldrich (St. Louis, MO, USA). Sodium borohydride was purchased from ACROS ORGANICS (Morris Plains, NJ, USA). Sodium hydroxide was ordered from Mallinckrodt Chemicals (Hampton, NJ, USA). Tetramethyl benzidine (TMB) substrate solution was obtained from Moss Inc. (Pasadena, MD, USA). Streptavidin poly-horseradish peroxidase (SA-HRP, Catalog No. 21130) and Sulfo-NHS-LC-Biotin were ordered from ThermoFisher Scientific (Rochester, NY, USA). Anti-spike protein antibody (Catalog No. 40150-R007) was obtained from Sino Biological, Inc. (Chesterbrook, PA, USA). Anti-nucleocapsid protein antibody (Catalog No. ABIN6952908) was purchased from Antibodies-online Inc. (Pottstown, PA, USA). Heat-inactivated SARS-CoV-2 (ATCC^®^ VR-1986HK™) was obtained from ATCC (Manassas, VA, USA). Nasal swab samples were obtained from Carle Foundation Hospital (Urbana, IL, USA) with approved protocols. All the listed chemicals and reagents were directly used as such in the experiments. Glassware used in the experiments was cleaned with freshly prepared aqua regia and rinsed with DI water six times.

### 2.2. Synthesis of Magnetic Nanoparticles

The magnetic nanoparticles used in the experiments are Fe_3_O_4_/Au core-shell nanoparticles. The Fe_3_O_4_ nanoparticles were synthesized according to the reported methods [33,34]. Typically, 3 mL of 1 M NaOH aqua solution was injected into 27 mL of DI water, which was then heated to boiling. After the addition of 2 mL of 0.4 M sodium citrate, 1 mL of 0.2 M ferrous chloride and 1 mL of 0.4 M of ferric chloride aqua solution was rapidly injected simultaneously with strong stirring. The resulting solution, the color of which changed instantly from colorless to black, was refluxed for 4 h. The obtained Fe_3_O_4_ nanoparticles were washed with ethanol and water three times, respectively, then dispersed in 10 of DI water.

The construction of Au shell around Fe_3_O_4_ was performed based on our previous reports [29,30]. Briefly, 920 μL of DI water was mixed with 80 μL of Fe_3_O_4_, and then sonicated for 10 min and centrifuged at 500 rpm for 100 min. The supernatant was added with 100 μL of 1% (*w*/*v*) HAuCl_4_ and the resulting mixture was sonicated for 10 min. Then, 200 μL of freshly prepared ice-cold NaBH_4_ was rapidly injected into the solution and the obtained solution was sonicated for 10 min. The generated Fe_3_O_4_/Au core-shell magnetic nanoparticles were washed with DI water and centrifuged 3 times and stored at 4 °C until modification.

### 2.3. Modification of Magnetic Probes

The modification of magnetic probes was based on Fe_3_O_4_/Au core-shell magnetic nanoparticles with antibody conjugation, blocking with casein, and biotinylation, performed per our prior works with minor modification [29,30]. To 0.25 mL of magnetic nanoparticles solution, 0.25 mL of DI water was added, then 3.35 μL of 0.5 M sodium carbonate and 50 μL of 10 mM phosphate buffer (pH = 7.4) was mixed with the diluted magnetic nanoparticle solution. Then, 5 μL of 1 mg/mL antibody was added to the obtained solution and shaken for 2.5 h. To this solution, 55 μL of 5% casein in 10 mM phosphate buffer was added and the resulting solution was shaken for 1 h. The antibody-modified magnetic nanoparticles were centrifuged and washed with 500 μL of 10 mM PBS 3 times and redispersed in 500 μL of 10 mM PBS. Then, 5 μg of sulfo-NHS-LC-biotin was injected and the solution was shaken for 1 h, and 50 μL of 5% casein was added and shaken for 1 h. The obtained magnetic nanoparticle probes were centrifuged and washed with 50 μL of 10 mM PBS 3 times and then redispersed in 50 μL of 0.5% casein in 10 mM PBS solution. The magnetic probes were stored at 4 °C until further use.

### 2.4. Preparation of Lateral Flow Strips

The LF strips used for the test were assembled by 2.5 cm of nitrocellulose (NC) membrane (90CNPH-N-SS40 from mdi Membrane Technologies, Harrisburg, PA, USA), 2.5 cm of absorbent pad (Grade 17 Chr Cellulose Chromatography Papers from GE Healthcare Life Sciences, MA, USA), 1.1 cm of conjugate pad (Grade 6613H from Ahlstrom North America, GA, USA), and 1.7 cm of sample pad (Sample Pad Type GFB-R4 from mdi Membrane Technologies, Harrisburg, PA, USA) on plastic backing cards (mdi Membrane Technologies, Harrisburg, PA, USA). A 0.2 cm overlap was provided between each part. At 1.3 cm from one end of the plastic backing card, the NC membrane was first fixed. The absorbent pad was attached at one side of the NC membrane and the conjugate pad and sample pad were attached at the other side. The assembled card was cut into stripes with 0.5 cm width.

### 2.5. Test of Virus in PBS Buffer and in Saliva

The antibody was conjugated to the LF strips by dropping 0.33 μL of 1 mg/mL antibody stock solution to the NC membrane and the strips were dried at 37 °C for 1 h. The SARS-CoV-2 virus positive control was diluted to serial concentrations with 10 mM PBS. To 150 μL of PBS samples, magnetic probes were added and incubated for 15 min. One microliter of 10 μg/mL SA-HRP was mixed with the solution and then loaded onto the LF strips. After 13 min of sample flow, the LF strip was washed with 60 μL of DI water with crossflow twice. Then, 60 μL of TMB substrate solution was applied followed by a 5 min incubation for colorimetric signal generation. The LF strip was washed again with 60 μL of water to stop the HRP catalyzed reaction and the colorimetric product beyond the test zone. The LF strip was then washed with 60 µL DI water. The resulting LF strips were imaged and the data processed with ImageJ (National Institutes of Health, Bethesda, MD, USA). After normalization for the brightness and contrast of the images, the quantified normalized color signal results were obtained with the grayscale value of the region with the deepest color at the dots minus the average grayscale value of the blank region of the strip. Error bars represent the deviation of the colorimetric results from replications. Human saliva was inoculated with positive control SARS-CoV-2 at serial dilutions utilizing the same protocol to validate the proposed detection method in clinical samples.

### 2.6. Test of Clinical Samples

We also obtained nasal swab samples from the Carle Foundation Hospital and the samples were stored in Puritan UniTranz-RT collection tubes at −80 °C before use. The clinical samples were heated at 65 °C for 30 min then cooled to room temperature. The samples were then tested with the same protocol. All the nasal swab samples were initially processed and inactivated in BSL-2 lab following the biosafety guideline from CDC [35].

## 3. Results and Discussion

**Scheme 1:**
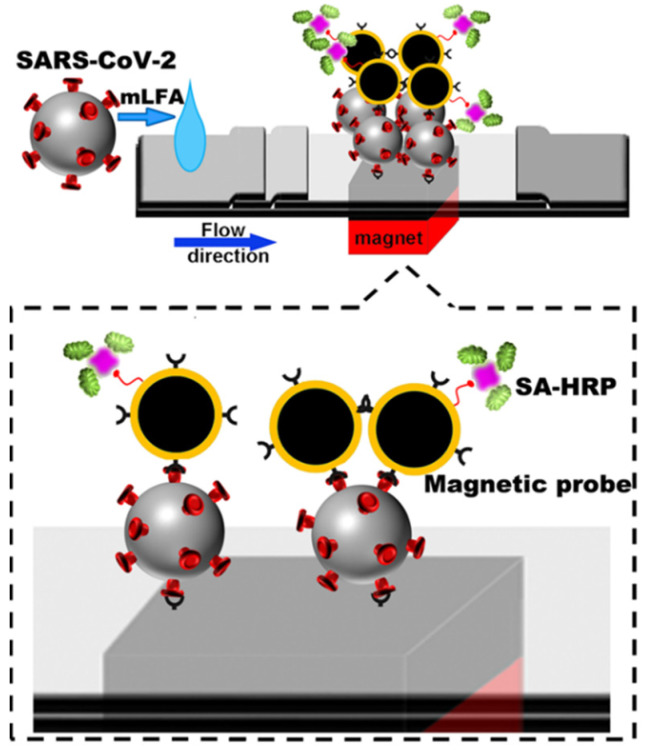
Schematic of mLFA detection of the SARS-CoV-2.

To recognize the SARS-CoV-2 virus, the anti-spike protein antibodies were conjugated to magnetic nanoparticles, which were then blocked with casein to reduce non-specific interaction and biotinylated to conjugate SA-HRP for colorimetric signal amplification. The obtained magnetic probes were used to label the target SARS-CoV-2 virus in the samples, which were mixed and incubated before loading the LF strip. As shown in Scheme 1, the sample solution was then loaded onto the LF strip and an external magnet was placed beneath it. During the mLFA process, the magnetic probes enable the magnetic focus, which concentrates the magnetic-probe-labeled SARS-CoV-2 virus at the signal generation zone as described in our prior investigation [30]. The magnetic force between the magnetic-probe-labeled SARS-CoV-2 virus and the magnet placed in the micro-channels in the LF strips help concentrate the labeled target viruses in the test zone. The magnetic force also focuses the magnetic probe-labeled targets close to the surface of the microchannels where the capture antibodies are immobilized. The movement of the labeled virus is significantly reduced to allow for a longer interaction time between capture antibody and target SARS-CoV-2. The increased local concentration of labeled SARS-CoV-2 in the capture antibody region and the interaction time greatly improves the capture efficiency thus resulting in improved detection sensitivity. The HRP linked to the probes generates a visible blue dot on the LF strip in the colorimetric amplification process with TMB substrate solution, enabling naked-eye recognition of the target SARS-CoV-2 without any additional instruments. The whole mLFA detection procedure could be completed within 50 min with only an LF strip and reusable magnet and without the need for any instrumentation.

**Figure 1:**
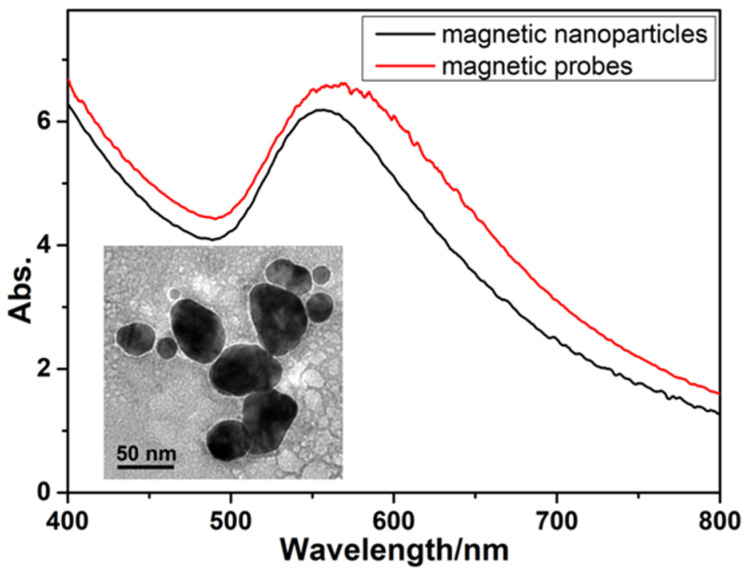
UV–vis spectra of magnetic nanoparticles and magnetic probes. Inset image is the TEM image of magnetic nanoparticles.

The magnetic probes are the core of the mLFA, which consists of Fe_3_O_4_ core/Au shell nanostructures. The Fe_3_O_4_ cores provide a strong magnetic response while the Au shells facilitate further modification and helps in the reduction of Fe_3_O_4_ oxidization. The morphology of the magnetic nanoparticles is illustrated by TEM as shown in the inset image in Figure 1. It can be seen that the magnetic nanoparticles are around 30–80 nm. As described in the experimental section, the generation of Au shells is a rapid process thus the morphology of the magnetic particles is not uniform. The extinction of nanostructures is highly influenced by the surface plasmon resonance; thus, the surface property change could induce a peak change in the extinction spectra of nanostructures. It can be seen in Figure 1 that a redshift and an increase in peak width could be observed in the extinction spectrum from magnetic probes compared with the magnetic nanoparticles, which could be assigned to antibody conjugation and blocking.

**Figure 2:**
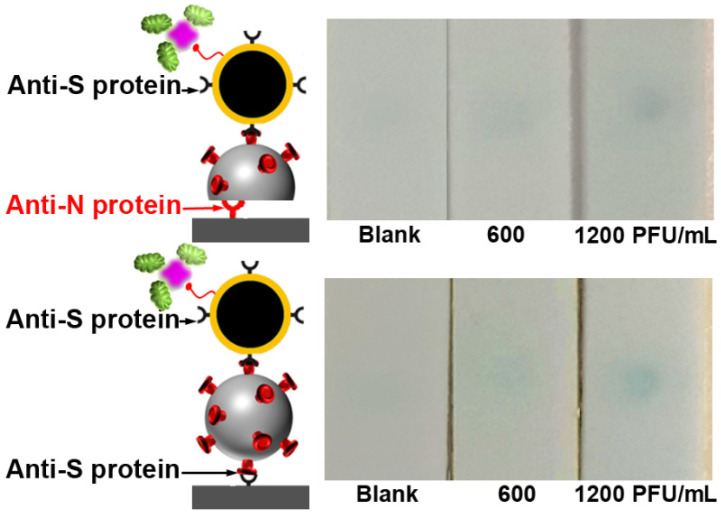
mLFA detection results with anti-spike protein antibody-modified magnetic probes on LF strips immobilized with anti-spike protein antibodies and anti-nucleocapsid protein antibodies, respectively.

To validate the detection capability of our mLFA, the heat-treated SARS-CoV-2 diluted in PBS buffer were tested. The magnetic nanoparticles were modified with anti-spike protein antibodies, while the LF strips were immobilized with anti-spike and anti-nucleocapsid protein antibodies, respectively. With these two types of LF strips, the SARS-CoV-2 diluted in PBS buffer were tested and the results are as shown in Figure 2. It can be seen that on the LF strips both the antibodies could capture the labeled target SARS-CoV-2 to generate clear blue dots for the recognition of the presence of the target virus. Compared with that on the strips immobilized with anti-spike protein antibodies, the color density of the dots is deeper on the LF strips immobilized with anti-nucleocapsid protein antibody when the samples with the same concentration of SARS-CoV-2 were tested. The nucleocapsid proteins are located within the SARS-CoV-2 virus, therefore the dots that appeared on the LF strips immobilized with anti-nucleocapsid protein antibody suggested that there were broken virus fragments in the heat-treated SARS-CoV-2 samples. The reported direct RT-PCR test with heat-treated SARS-CoV-2 indicated that the heat treatment would rupture the SARS-CoV-2 into fragments [36]. The dots obtained on the LF strips with anti-nucleocapsid protein antibodies should be attributed to these fragments, and the deeper color of the dots implied that the fragments labeled with anti-spike protein antibody-modified magnetic probes can be easily captured by anti-nucleocapsid protein antibodies than anti-spike antibodies on LF strips. However, an mLFA with anti-spike protein antibodies conjugated onto the magnetic probes and LF strips could enable the detection of samples without heat treatment, which could extend the application of the mLFA for SARS-CoV-2 detection to facilitate on-site monitoring. In the following sections, the mLFA detection was carried out with anti-spike protein antibodies conjugated magnetic probes and LF strips.

**Figure 3:**
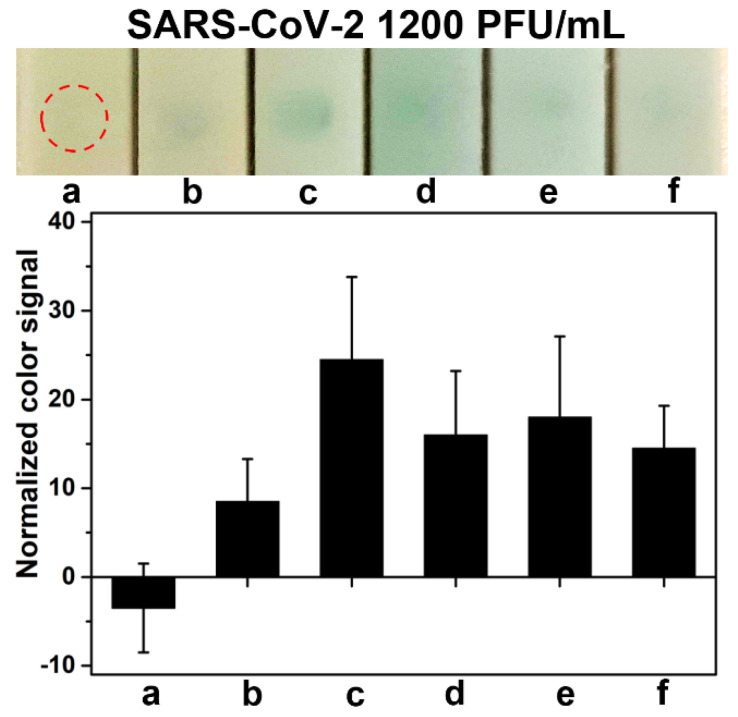
Detection results and the normalized colorimetric signal from 1200 PFU/mL of SARS-CoV-2 in PBS buffer with 1.5 picomoles (a, 142.8 %RSD), 3.0 picomoles (b, 56.5 %RSD), 7.5 picomole (c, 37.9 %RSD), 15 picomoles (d, 45.0 %RSD), 30 picomoles (e, 50.6 %RSD), and 60 picomoles (f, 33.1 %RSD) of magnetic probes (*n* = 3) The red-dotted circle overlaid on the image indicates the position where the anti-spike antibody was applied on the LF strip for the test with 1.5 picomoles of magnetic probes.

Because the antibodies modified on the magnetic probes are anti-spike protein antibodies and there are multiple spike proteins on the shell of SARS-CoV-2, it is possible that there is more than one magnetic probe labeled on a virus. Considering the size of the SARS-CoV-2 as 70–90 nm, the labeling of multiple magnetic probes at 30–80 nm would induce a steric hindrance on the capture antibody on an LF strip to capture the labeled target SARS-CoV-2 to affect detection sensitivity. Therefore, more magnetic probes would increase the labeling ratio in the incubation while any further increase in the number of magnetic probes would influence the capture efficiency with capture antibodies immobilized onto the LF strips. To demonstrate the relationship between colorimetric signal intensity and the number of added magnetic probes, samples with 1200 PFU/mL of SARS-CoV-2 in PBS buffer were tested with a serial number of magnetic probes and the results are as shown in Figure 3. It can be seen that the blue dots become more obvious when the number of magnetic probes increase from 1.5 picomoles to 7.5 picomoles. However, when increasing the amount of the magnetic probes more than 7.5 picomoles, the color of the dots tends to become weaker. The clearest dots were obtained with 7.5 picomoles of magnetic probes. Compared to the bacteria tested with the mLFA [29], the smaller size of the SARS-CoV-2 benefits the magnetic control of the labeled target virus in the magnetic focus enhancement. Therefore, with the optimal level of magnetic probes, recognition of 1200 PFU/mL of SARS-CoV-2 with the naked eye is possible due to the generation of blue dots on the LF strips.

**Figure 4:**
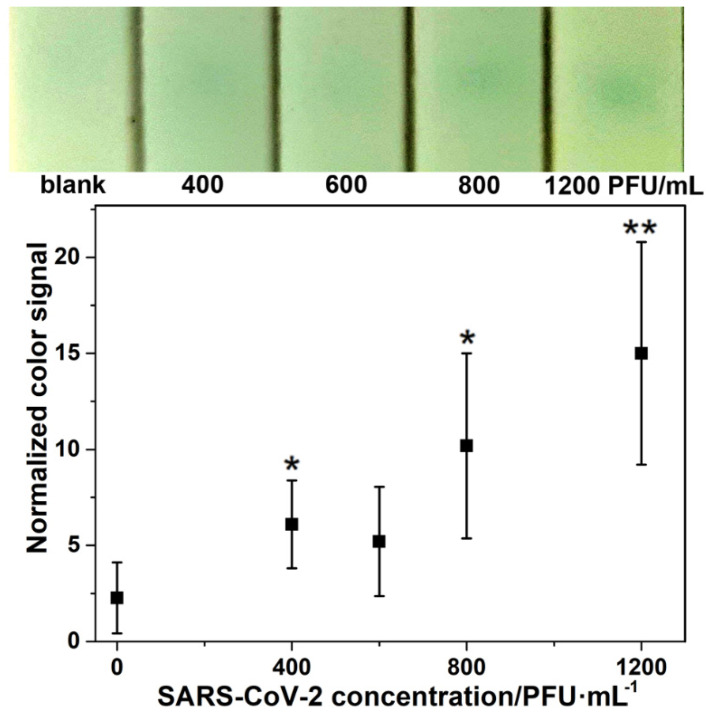
Detection results and the normalized colorimetric signal from blank (81.4 %RSD), 400 PFU/mL (37.5 %RSD, *p*-value 0.087 vs. blank marked as * in Figure 4), 600 PFU/mL (54.7 %RSD, *p*-value 0.21 vs. blank), 800 PFU/mL (47.4 %RSD, *p*-value 0.057 vs. blank marked as * in Figure 4), and 1200 PFU/mL (38.6 %RSD, *p*-value 0.022 vs. blank marked as ** in Figure 4) of SARS-CoV-2 in PBS buffer (*n* = 3).

Under the optimized test condition, SARS-CoV-2 at a serial concentration in PBS buffer was detected with the mLFA. An image of typical detection results is shown in Figure 4. It can be seen that no dot is present on the LF strip with blank samples. With samples containing 400 PFU/mL or 600 PFU/mL SARS-CoV-2, only very light dots can be noted on the LF strip. When the concentration of SARS-CoV-2 increased to 800 PFU/mL or higher, the dots on the LF strips can be easily recognized with the naked eye. The color density of the dots was quantified with the normalized color signal. The normalized color signal indicates the color difference between the dots and the background. It can be seen that the normalized color signal from the samples containing 400 PFU/mL and 600 PFU/mL is similar, while in samples with 600 PFU/mL to 1200 PFU/mL the normalized color signal exhibits a marked increase. The deviation of the normalized signal could be attributed to the SARS-CoV-2 fragments at different sizes after heat treatment: smaller fragments do not contain enough spike proteins on the shell for labeling and capture, which may give rise to a decrease in the normalized color signal. The deviation in the quantified signal makes the quantitative detection difficult, but the qualitative determination of SARS-CoV-2 with the naked eye is possible based on clear dots that appear on the LF strips. To demonstrate the magnetic focus enhancement in the proposed mLFA, we performed the same detection procedure without the magnet in the LF strip. As shown in Figure S2, a signal (depicting no dot) is not visible on the LF strip for a target concentration of 1200 PFU/mL of SARS-CoV-2 in PBS buffer, though the non-specifically bound magnetic probes and HRP on the strip induce a background on the strips. Furthermore, blank samples and 1200 PFU/mL of SARS-CoV-2 in PBS were also tested with a typical conventional LFA with gold nanoparticles (GNPs) modified with anti-spike antibody as GNP probes without colorimetric amplification. The test results are shown in Figure S2. It can be noted that there is no visible signal from GNP probes, indicating that the conventional LFA may not be able to recognize SARS-CoV-2 at such low concentrations.

**Figure 5:**
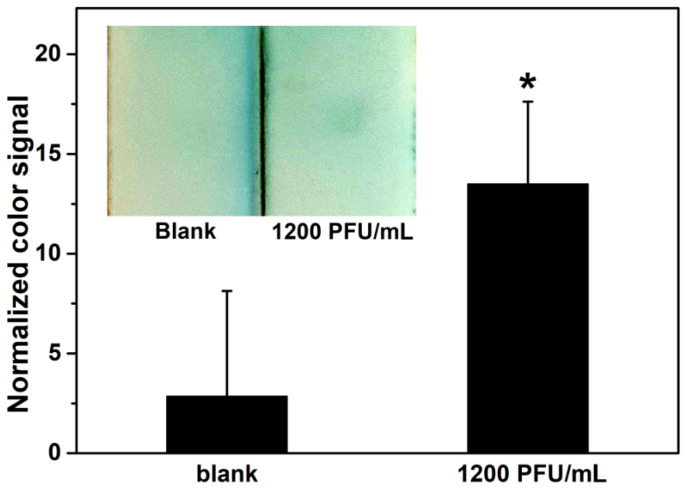
Normalized colorimetric signal from mLFA detection of uninoculated saliva (184.1% RSD) and 1200 PFU/mL (30.6 %RSD, *p*-value 0.051 vs. uninoculated saliva marked as * in Figure 5) of SARS-CoV-2 inoculated saliva (*n* = 3). The inset image shows typical detection results with saliva samples.

To investigate the mLFA detection of SARS-CoV-2 in saliva samples, human saliva inoculated with SARS-CoV-2 at a final concentration of 1200 PFU/mL was tested and the results are shown in Figure 5. Compared to PBS buffer, saliva is more complex and viscous and flows much slower in LF strips. Although the slower flow speed could increase the capture time of labeled SARS-CoV-2, the non-target components in the saliva solution could affect detection performance and specificity when using saliva samples. In the inset image of Figure 5, a clear blue dot on the LF strip is noted for a concentration of 1200 PFU/mL of SARS-CoV-2 inoculated saliva sample, while no dot is observed in the uninoculated saliva. Due to the non-target components and reduced flow, there is more unreacted magnetic probes and SA-HRP left in the LF strips, resulting in a stronger background compared to that with PBS samples. It is expected that these non-target components would influence the capture of magnetic probes labeled SARS-CoV-2 with the antibodies immobilized on LF strips. Thus, the dots from the 1200 PFU/mL SARS-CoV-2 saliva samples are not as clear as that from PBS samples. The normalized color signals from the blank and 1200 PFU/mL of SARS-CoV-2 in saliva were also plotted in Figure 5. A more significant deviation should be attributed to the unreacted magnetic probes and SA-HRP on the LF strips. The average normalized color signal from 1200 PFU/mL of SARS-CoV-2 in saliva is slightly weaker than that from the PBS samples, suggesting the influence of non-target components in saliva on the capture and background color on the LF strips. Compared with the value of normalized color signals, the appearance of blue dots is a better indicator of the presence of SARS-CoV-2. Based on the dots on the strips in the mLFA test, nasal swab samples in Puritan UniTranz-RT collection tubes were tested with the proposed mLFA, and 66.7% sensitivity and 100% specificity were achieved.

## 4. Conclusions

Herein an mLFA detection strategy for monitoring SARS-CoV-2 was proposed. The mLFA is a lateral flow-based analytical technique that requires only LF strips and a small magnet for highly sensitive detection of the target virus. The detection procedure can be performed within 50 min and the colorimetric results will allow naked-eye monitoring of the targets. The magnetic focus enhancement and enzymatic amplification of the colorimetric signal enables the proposed mLFA sensor to detect as low as 400 PFU/mL of SARS-CoV-2 in PBS buffer. The mLFA test also shows good detection sensitivity in saliva samples, where 1200 PFU/mL of SARS-CoV-2 was detected. With nasal swab samples, the proposed mLFA technology provided 66.7% sensitivity and 100% specificity. The short test time, low cost, simplicity, and practicality of the mLFA technique makes it an affordable test for use in low resource settings.

## Supplementary Materials

The following is available online at https://www.mdpi.com/article/10.3390/bios11120488/s1, Figure S1: Images of LF strips from blank sample and 1200 PFU/mL of SARS-CoV-2 in PBS recorded from time course experiments; Figure S2: Detection results of blank and 1200 PFU/mL of SARS-CoV-2 in PBS buffer with a conventional LFA with GNP probes and mLFA performed without magnet during the test.
