# Supplementary material for: Paper-Based Test for Rapid On-Site Screening of SARS-CoV-2 in Clinical Samples
Source: Biosensors (Basel). 2021 Nov 30;11(12):488. doi: 10.3390/bios11120488 (PMC8699507; doi:10.3390/bios11120488)
Supplement: Supplementary file 1 [file biosensors-11-00488-s001.zip › biosensors-1449374-supplementary.pdf]

biosensors

MDPI

Paper-Based Test for Rapid On-Site Screening of SARS-Cov-2 in Clinical Samples

Wen Ren [1,2] and Joseph Irudayaraj [1,2,3,4,*]

[1] Department of Bioengineering, University of Illinois at Urbana-Champaign, Urbana, IL 61801, USA; wenren@illinois.edu
[2] Biomedical Research Center in Mills Breast Cancer Institute, Carle Foundation Hospital, Urbana, IL 61801, USA
[3] Micro and Nanotechnology Laboratory, University of Illinois at Urbana-Champaign, Urbana, IL 61801, USA
[4] Cancer Center at Illinois (CCIL), University of Illinois at Urbana-Champaign, Urbana, IL 61801, USA
* Correspondence: jirudaya@illinois.edu

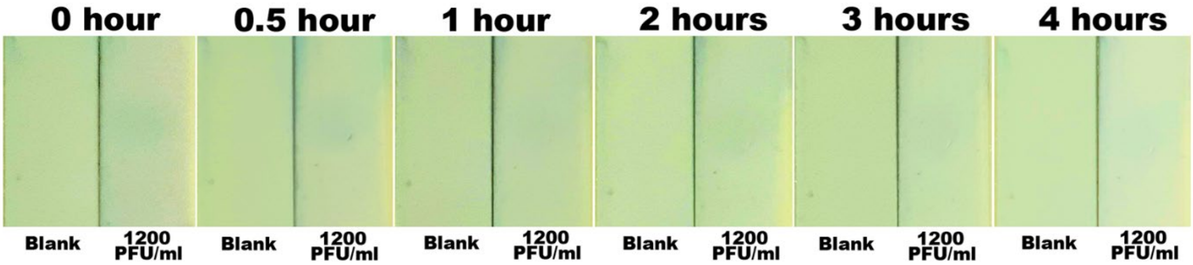

Figure S1. Images of LF strips from blank sample and 1200 PFU/ml of SARS-CoV-2 in PBS recorded from time course experiments. After 30 min, the dot on the LF strip from 1200 PFU/ml is compared to that recorded just after the test. When the time increases, the color of the dot became more vague and more diffuse. After 4 hours, the dot on the LF strip was very difficult to discern. Therefore, to minimize the influence of the appearance of the dot with time and to reduce the test time, test results should be noted after the mLFA procedure.

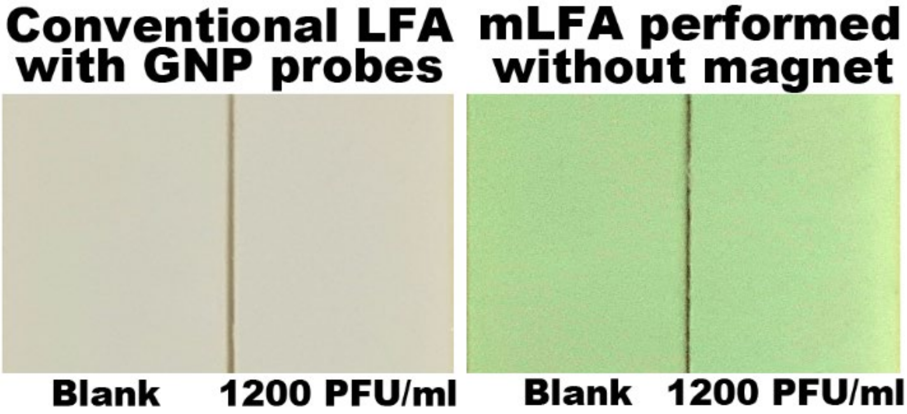

Figure S2. Detection results of blank and 1200 PFU/ml of SARS-CoV-2 in PBS buffer with a conventional LFA with GNP probes and mLFA performed without magnet during the test.
